# Sample Source and Cryopreservation Affect Flow-Cytometric Profiling of Immune-Escape-Related Markers on AML Blasts

**DOI:** 10.3390/cancers18162559

**Published:** 2026-08-09

**Authors:** Sven Richter, Benedikt Leinauer, Alicia Thoma, Ann-Kristin Schmälter, Johanna Waidhauser, Christoph Schmid, Andreas Rank, Phillip Löhr

**Affiliations:** Department of Hematology and Oncology, University Medical Center Augsburg, 86156 Augsburg, Bavaria, Germany; benedikt.leinauer@uk-augsburg.de (B.L.); alicia.thomca@uk-augsburg.de (A.T.); ann-kristin.schmaelter@uk-augsburg.de (A.-K.S.); johanna.waidhauser@uk-augsburg.de (J.W.); christoph.schmid@uk-augsburg.de (C.S.); andreas.rank@uk-augsburg.de (A.R.); phillip.loehr@uk-augsburg.de (P.L.)

**Keywords:** AML, flow cytometry, cryopreservation, CXCR4, immune escape, RFI, MFI

## Abstract

**Background/Objectives:** Flow-cytometric readouts in acute myeloid leukemia (AML) may depend on specimen source and processing. We assessed whether sample source (peripheral blood [PB] vs. bone marrow [BM]) and cryopreservation influence immune-evasion-related markers on AML blasts. **Methods:** Paired PB and BM samples from 19 adults were analyzed fresh and after cryopreservation. Viable CD45^dim^ singlets were assessed for ULBP1-6, MICA/B, CD34, CD44, CD70, CD80, and CD184 using median fluorescence intensity (MFI) and relative fluorescence index (RFI). Paired comparisons used two-sided Wilcoxon signed-rank tests with Benjamini–Hochberg correction across six markers within each endpoint and comparison. Discordance was also assessed using a predefined conventional 20% reference threshold. **Results:** After adjustment, RFI did not differ between fresh and cryopreserved samples within PB or BM, or between fresh PB and BM. In cryopreserved samples, PB and BM differed for CD70 (q = 0.042) and ULBP1-6 (q = 0.028). Cryopreservation increased MFI for CD184 and ULBP1-6 in PB and BM and for MICA/B in PB; no PB-BM MFI difference remained significant. Threshold-based classification was discordant for CD34, CD44, CD80, CD184, MICA/B, and ULBP1-6 in 5.3–38.9% of pairs, whereas CD70 showed none. **Conclusions:** Cohort-level differences were limited to specific markers and conditions, while patient-level threshold discordance persisted. Sample source and cryopreservation should therefore be considered when interpreting threshold-based classifications.

## Simple Summary

Flow cytometry is central to acute myeloid leukemia (AML) assessment, but bone marrow (BM) is sometimes replaced by peripheral blood (PB), and samples may be cryopreserved. We compared paired PB and BM specimens from 19 patients, analyzed fresh and after cryopreservation, for seven blast-cell markers. After correction for multiple testing, relative fluorescence index differences remained only between cryopreserved PB and BM for CD70 and ULBP1-6. Cryopreservation altered median fluorescence intensity of CD184 and ULBP1-6 in both compartments and MICA/B in PB, while no PB-BM MFI differences remained. At a predefined conventional 20% reference threshold, 5.3–38.9% of paired measurements for all markers except CD70 were classified differently. Thus, both sample source and cryopreservation can influence selected marker readouts, and threshold-based classifications should be interpreted cautiously.

## 1. Introduction

Acute myeloid leukemia (AML) is a heterogeneous hematologic malignancy characterized by the clonal expansion of immature myeloid progenitors within the bone marrow (BM) and peripheral blood (PB). Multiparameter flow cytometric immunophenotyping plays a pivotal role in the diagnostic workup of AML, facilitating the rapid detection and characterization of leukemic blasts [1]. Although subclassification and prognostication in AML rely on genetic and cytogenetic findings, flow cytometry remains indispensable for lineage assignment, detecting leukemia-associated immunophenotypes, and measurable residual disease monitoring [2,3]. However, two pre-analytical issues are particularly relevant to the present study:

**Cryopreservation may alter marker expression and cell viability.** In clinical and research settings, the cryopreservation of blood and bone marrow samples is commonly employed to facilitate sample handling, long-term storage, and multicenter collaboration [4]. Although this technique is indispensable in large-scale studies and biobanking, the freeze–thaw process may alter cell viability, membrane integrity, and the detectability of surface antigens [5]. For instance, reduced expression of CD62L and CD45RA has been observed after cryopreservation, potentially compromising downstream analyses [6]. Conversely, some studies reported no significant differences for selected markers, suggesting that the effects of cryopreservation are not universal. These issues raise concerns about the comparability of fresh and cryopreserved samples, especially in immunophenotyping studies [5,7,8].

**Marker expression may differ between peripheral blood and bone marrow.** In clinical diagnostics, peripheral blood is frequently used as a surrogate for bone marrow, particularly in cases with high circulating blast counts or when bone marrow aspiration is not feasible (e.g., in punctio sicca) [9]. Although most routinely assessed markers (e.g., CD4 or CD8, as well as markers utilized for AML diagnostics like CD13 or CD33) [10] show comparable expression between PB and BM blasts, less well-characterized or functionally dynamic antigens (including immune-escape-related markers) may differ between compartments, potentially complicating data interpretation, particularly when combined with cryopreservation effects [11,12].

To date, most investigations into cryopreservation effects have focused on well-established surface markers in peripheral blood mononuclear cells (PBMC) or on conventional leukocyte populations in whole blood. Studies comparing fresh and cryopreserved samples have shown generally good comparability for several routinely used markers and major leukocyte subsets [13,14]. However, preservation is not uniform across all antigens, and altered detectability has been reported for selected surface markers [6,15]. Thus, findings obtained for conventional markers cannot necessarily be extrapolated to less well-characterized, functionally regulated antigens. In contrast, data on immune escape-related surface antigens in hematologic malignancies remain scarce, particularly in AML, where these markers are often heterogeneously expressed, and their stability under different sampling and processing conditions remains uncertain [8,16].

In recent years, immune escape has emerged as a key mechanism of disease persistence and therapy resistance in AML, in particular in the context of allogeneic stem cell transplantation [17,18,19]. We selected six functionally relevant surface antigens with established or potential therapeutic relevance, while CD34 served as a conventional reference marker. CD34 is associated with immature progenitor populations [2]; CD44 mediates adhesion and interactions with the bone marrow niche [20]; CD70–CD27 signaling contributes to leukemic stem cell maintenance [21]; CD80 regulates T-cell costimulation through CD28 and CTLA-4 [22]; CD184/CXCR4 controls bone marrow homing and retention [23,24]; and MICA/B and ULBP1-6 are stress-inducible NKG2D ligands involved in NK-cell recognition [16]. CD44, CD70, and CXCR4 have been therapeutically targeted in AML studies, whereas CD80 and NKG2D-ligand modulation have been explored in experimental immunotherapeutic approaches [16,20,21,22,23,24]. Because these markers reflect adhesion, trafficking, costimulation, and cellular stress responses, their surface detectability may be more context-dependent than that of conventional lineage-defining markers. Accordingly, sample collection and storage remain critical pre-analytical factors when PB and BM or fresh and cryopreserved samples are compared [4,5,8,10,13,14,15].

In this prospective study, we investigated immune-escape-related markers on leukemic blasts across sampling compartments (bone marrow and peripheral blood) and processing conditions (fresh and cryopreserved) to assess their comparability for translational analyses.

## 2. Materials and Methods

### 2.1. Study Design and Population

This prospective, cross-sectional laboratory study was conducted at University Hospital Augsburg and enrolled 19 adults with acute myeloid leukemia. Of these, 17 were enrolled at initial diagnosis, one at relapse, and one with progressive disease. Paired peripheral blood (PB) and bone marrow (BM) aspirate samples were collected from each patient. All patients had more than 20% blasts in BM and measurable circulating blasts in PB during routine diagnostic assessment. Samples were processed in four matched conditions per patient: fresh PB, cryopreserved PB, fresh BM, and cryopreserved BM.

### 2.2. Sample Collection, Cryopreservation, and Thawing

PB was drawn into K_3_-EDTA tubes. BM aspirates were collected using standard syringes preloaded with 3 mL of EDTA solution to prevent clotting.

Fresh samples were processed within 24 h of collection, beginning with the isolation of PBMCs and BMMCs by density gradient centrifugation (Pancoll human, density 1.077 g/mL, PanBiotech (Aidenbach, Germany)), followed by washing with PBS. Cryopreservation was performed using the Cryo™ ABC Media Kit (C.T.L., Cellular Technology Limited, Shaker Heights, OH, USA) according to the manufacturer’s protocol for the *Cryopreservation of PBMCs from Fresh Whole Blood*. The samples were subsequently stored at −80 °C for 24–48 h, in accordance with the standard short-term workflow of our institutional biobank, before thawing and flow-cytometric analysis.

For thawing, the cryovials containing PBMCs or BMMCs were incubated at 25 °C for 5–10 min, gently resuspended by inversion, and transferred into two 15 mL tubes containing 14 mL of room-temperature PBS (DPBS; Gibco™, Thermo Fisher Scientific, Grand Island, NY, USA). The tubes were gently inverted to mix, and the cell suspensions were centrifuged for 5 min at 1000× *g*. The supernatant was discarded, and the cell pellet was resuspended in PBS. Finally, cell count and viability were determined and adjusted as necessary.

### 2.3. Immunophenotypic Staining and Flow Cytometry

Cell suspensions were incubated with a panel of monoclonal antibodies targeting ULBP1-6, MICA/B, CD34, CD44, CD45, CD70, CD80, and CD184. The viability dye 7-AAD was used to exclude nonviable cells. Antibodies were purchased from Beckman Coulter (Brea, CA, USA), BioLegend (San Diego, CA, USA), and R&D Systems (Minneapolis, MN, USA), and clone information is provided in Supplementary Table S5.

Following staining, the cell suspensions were treated with VersaLyse Lysing Solution (Beckman Coulter) and IOTest 3 Fixative Solution (Beckman Coulter) according to the manufacturer’s instructions and subsequently washed with PBS.

Data acquisition was performed on a Navios EX flow cytometer (Beckman Coulter). To ensure data quality, flow rate, laser performance, and event dispersion were checked daily using Flow-Check Pro Fluorospheres (Beckman Coulter). AutoSetup procedures were routinely repeated and documented to generate automated compensation matrices. Detector voltages and compensation settings for all fluorescence channels were specifically verified using compensation beads for the multiparameter flow cytometry panel applied in this study. Plausibility controls using reference material from healthy donors were performed repeatedly, and identical samples were regularly measured as internal standards to monitor assay consistency.

### 2.4. Gating Strategy

Data compensation was performed using Kaluza analysis software (Beckman Coulter, v1.1). The compensated data were then transferred to Cytobank (Beckman Coulter, v11.0) using a custom in-house script written in R. Within Cytobank, a data quality check was performed to identify and eliminate technical outliers. Singlets were identified based on forward scatter height versus area, followed by exclusion of nonviable cells using 7-AAD staining. CD45^dim^ cells were subsequently gated to isolate the blast population, which was further analyzed with the respective markers. Fluorescence minus one (FMO) controls were used to define the gating boundaries for each fluorescence channel. The CD45^dim^ gate was manually set for each measurement to encompass the blast population; the flow-cytometric blast proportion was calculated as 100 × CD45^dim^ viable events divided by all viable singlet events (Supplementary Figure S1).

### 2.5. Software

Data processing and visualization were performed in R (v4.5.3) using tidyverse (v2.0.0), ggstatsplot (v0.13.5), readxl (v1.4.5), scales (v1.4.0), and rlang (v1.1.7), and in Python (v3.13.5) using pandas (v2.2.3), NumPy (v2.1.3), Matplotlib (v3.10.0), seaborn (v0.13.2), and SciPy (v1.15.3).

### 2.6. Statistical Analysis

Paired comparisons between sample compartments and processing conditions were performed using the Wilcoxon signed-rank test. All tests were two-sided. To account for multiple testing, *p* values were adjusted using the Benjamini–Hochberg procedure separately for each endpoint (RFI and MFI) and paired comparison across the six investigated markers. For positive-cell proportions, adjustment was performed separately within each paired comparison across all seven markers, including CD34. Adjusted *p* values are reported as q-values, and statistical significance was defined as q < 0.05. RFI was calculated as the MFI of the marker-positive blast population divided by the MFI of the corresponding marker-negative reference population within the same sample. Thus, RFI represents a background-normalized measure of fluorescence intensity. Concordance of paired RFI measurements was quantified on the log2 scale using Lin’s concordance correlation coefficient (CCC) [25] with percentile-bootstrap 95% confidence intervals based on 5000 resamples. CCC estimates were interpreted descriptively because no clinically acceptable threshold for agreement had been prespecified. For the primary exploratory analysis of discordance in dichotomized marker classification, a 20% reference threshold was used. This value reflects a historical convention in acute leukemia immunophenotyping [26,27] and should not be interpreted as a fixed, clinically validated, or marker-specific cutoff for the antigens investigated. A pair was classified as discordant when one measurement was below the respective threshold and the corresponding measurement was at or above it. To assess threshold sensitivity, the analysis was repeated using 10% and 30% reference thresholds.

## 3. Results

A total of 19 patients with acute myeloid leukemia (AML) were included in the analysis. The study cohort comprised 8 male and 11 female individuals, with a median age of 61.4 years (range 32–87 years). Of the 19 patients, 17 were enrolled at initial diagnosis, one at relapse, and one with progressive disease. According to the ELN 2022 risk classification, 4 patients were categorized as favorable, 3 as intermediate, and 12 as adverse. The median flow-cytometric blast proportions among viable singlets were 70.3% (range 2.3–95.7%) in fresh PB and 76.6% (4.9–97.9%) in cryopreserved PB, compared with 85.8% (35.1–96.5%) in fresh BM and 85.3% (27.6–97.8%) in cryopreserved BM. The median viability, calculated as the proportion of 7-AAD-negative events among singlets, was 98.1% (range 87.8–99.9%) in fresh PB, 90.3% (53.9–99.2%) in cryopreserved PB, 95.4% (76.9–99.8%) in fresh BM, and 84.5% (41.5–97.5%) in cryopreserved BM. FAB classification was M1 in 5 patients and M2 in 13 patients; it was unavailable for one patient because of punctio sicca. Patient-level ELN 2022 risk groups, FAB classification, and documented molecular findings are provided in Supplementary Table S4.

For each patient, both PB and BM samples were obtained. Each specimen was analyzed under two conditions: freshly processed or following cryopreservation, resulting in four datasets per patient. Expression levels of seven surface markers (ULBP1-6, MICA/B, CD34, CD44, CD70, CD80, CD184) were assessed across all conditions. Both MFI and RFI were measured.

Due to a technical artifact affecting ULBP1-6, CD70, CD80, and MICA/B in the fresh bone marrow sample of participant 15, all measurements for these markers from this participant were excluded across all four conditions to preserve the paired study design before downstream visualization and analysis.

### 3.1. Peripheral Blood: Fresh vs. Cryopreserved

**Figure 1:**
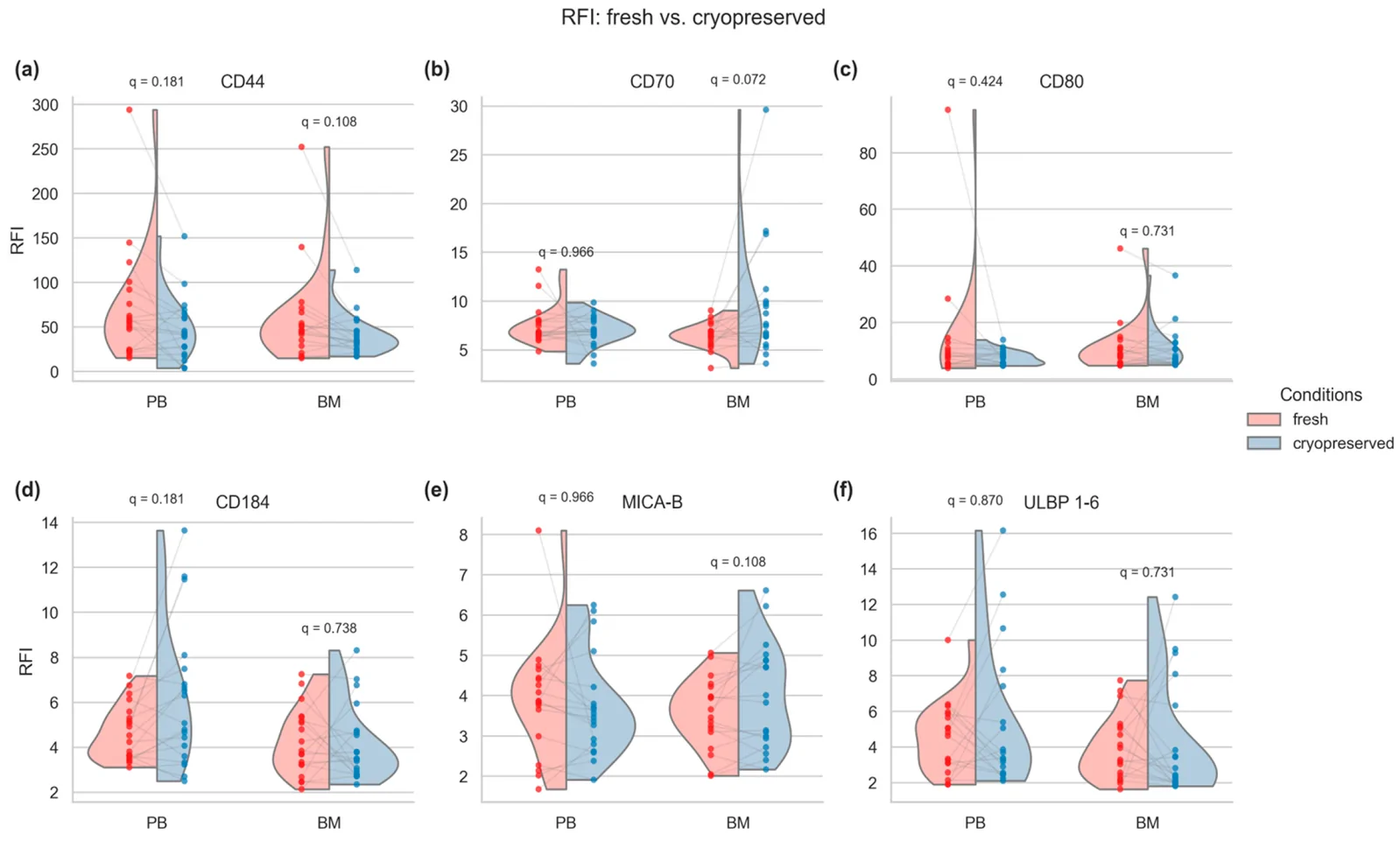
RFI fresh vs. cryopreserved. Relative fluorescence intensity (RFI) in fresh versus cryopreserved samples for (**a**) CD44, (**b**) CD70, (**c**) CD80, (**d**) CD184, (**e**) MICA/B, and (**f**) ULBP 1-6 in peripheral blood (PB) and bone marrow (BM). Lines connect paired samples from the same patient; statistics were performed using the Wilcoxon signed-rank test. Displayed q-values are Benjamini–Hochberg-adjusted *p*-values from two-sided Wilcoxon signed-rank tests. Adjustment was performed separately within each endpoint and comparison across the six investigated markers. Statistical significance was defined as q < 0.05.

After Benjamini–Hochberg adjustment, no statistically significant RFI differences were observed between fresh and cryopreserved PB samples for CD44 (q = 0.181), CD70 (q = 0.966), CD80 (q = 0.424), CD184 (q = 0.181), MICA/B (q = 0.966), or ULBP1-6 (q = 0.870) (Figure 1). MFI remained significantly higher after cryopreservation for CD184 (q < 0.001), MICA/B (q = 0.015), and ULBP1-6 (q = 0.002) (Supplementary Figure S2, Supplementary Table S1).

### 3.2. Bone Marrow: Fresh vs. Cryopreserved

After adjustment, no statistically significant RFI differences were observed between fresh and cryopreserved BM samples for CD44 (q = 0.108), CD70 (q = 0.072), CD80 (q = 0.731), CD184 (q = 0.738), MICA/B (q = 0.108), or ULBP1-6 (q = 0.731) (Figure 1). MFI remained significantly higher after cryopreservation for CD184 (q = 0.003) and ULBP1-6 (q < 0.001), whereas MICA/B did not retain statistical significance (q = 0.077) (Supplementary Figure S2, Supplementary Table S1).

### 3.3. Fresh Samples: Peripheral Blood vs. Bone Marrow

**Figure 2:**
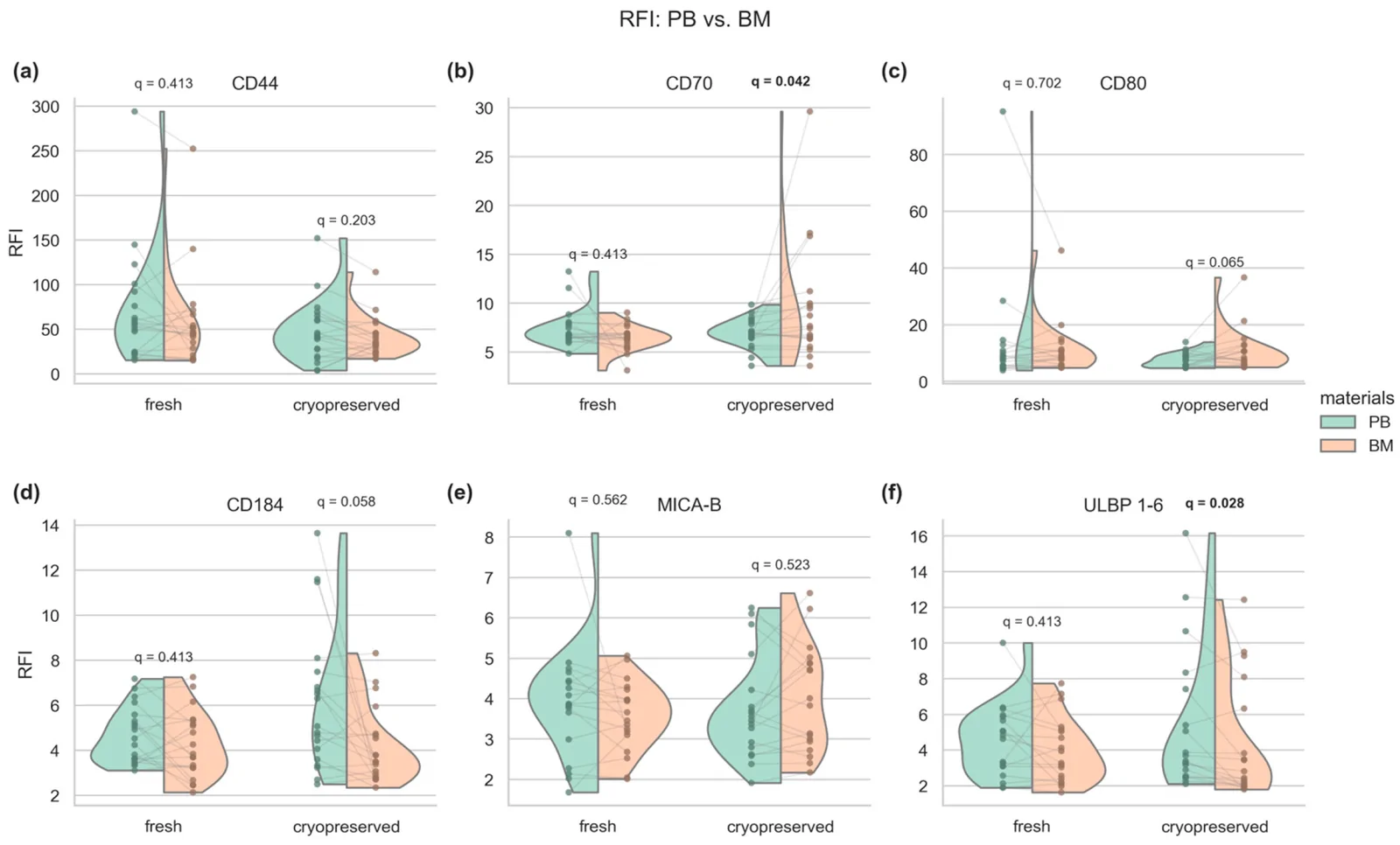
RFI in PB versus BM for (**a**) CD44, (**b**) CD70, (**c**) CD80, (**d**) CD184, (**e**) MICA/B, and (**f**) ULBP 1-6 in (1) fresh and (2) cryopreserved samples. Lines connect paired samples from the same patient; statistics were performed using the Wilcoxon signed-rank test. Displayed q-values are Benjamini–Hochberg-adjusted *p*-values from two-sided Wilcoxon signed-rank tests. Adjustment was performed separately within each endpoint and comparison across the six investigated markers. Statistical significance was defined as q < 0.05.

No statistically significant differences in RFI were observed between freshly processed PB and BM samples for CD44 (q = 0.413), CD70 (q = 0.413), CD80 (q = 0.702), CD184 (q = 0.413), MICA/B (q = 0.562), or ULBP1-6 (q = 0.413) (Figure 2). No MFI comparison between fresh PB and BM remained statistically significant after adjustment (all q ≥ 0.259; Supplementary Figure S3, Supplementary Table S1).

### 3.4. Cryopreserved Samples: Peripheral Blood vs. Bone Marrow

After adjustment, RFI values differed significantly between cryopreserved PB and BM samples for CD70 (q = 0.042) and ULBP1-6 (q = 0.028), while no statistically significant differences were observed for CD44 (q = 0.203), CD80 (q = 0.065), CD184 (q = 0.058), or MICA/B (q = 0.523) (Figure 2). No MFI comparison between cryopreserved PB and BM remained statistically significant after adjustment (all q ≥ 0.097; Supplementary Figure S3, Supplementary Table S1).

### 3.5. Concordance of RFI Measurements

Across the 24 marker-by-comparison analyses, Lin’s CCC estimates for log2-transformed RFI values ranged from −0.397 to 0.816, with wide bootstrap 95% confidence intervals for several comparisons (Supplementary Table S2). Point estimates varied across markers and conditions.

### 3.6. Discordance Relative to the 20% Reference Threshold

Across all four comparisons, the magnitude and direction of paired changes varied considerably between patients, as visualized in Figure 1 and Figure 2. To further characterize these patient-level differences, we assessed discordance relative to the predefined conventional 20% reference threshold. In paired percentage plots, discordant classification occurred when one measurement was below 20% and the corresponding measurement was at or above 20%. Discordant classification was observed across all four comparisons for CD34, CD184, MICA/B, and ULBP1-6. For CD44, discordance occurred only in the PB-versus-BM comparisons (5.3% for both fresh and cryopreserved samples), whereas CD80 showed discordance only for fresh PB versus fresh BM and for fresh versus cryopreserved PB (5.6% each). Across all markers and comparisons, nonzero discordance ranged from 5.3% to 38.9%. By contrast, no paired measurements showed discordant classification relative to the predefined conventional 20% reference threshold for CD70 in any comparison (Table 1, Supplementary Figure S4, Supplementary Figure S5). In sensitivity analyses, discordance was observed in 25 of 28 marker-by-comparison combinations at the 10% threshold, 20 of 28 at 20%, and 22 of 28 at 30%, with nonzero discordance rates of 5.3–44.4%, 5.3–38.9%, and 5.3–38.9%, respectively. Discordance occurred at all three thresholds in 18 of 28 combinations and at least one threshold in 27 of 28 combinations. The marker-specific pattern was threshold-dependent; for example, CD70 showed discordance in all four comparisons at 10% but none at 20% or 30% (Supplementary Table S3).

## 4. Discussion

Immune escape mechanisms are increasingly recognized as important in AML [17,19]. Therefore, the precise measurement of these markers is essential. Although peripheral blood and cryopreserved samples are commonly used as surrogates for fresh bone marrow, this approach has been systematically evaluated only for a limited number of mostly conventional markers [4,7,8]. In contrast, the validity of this approach for markers relevant to immune monitoring and immune escape remains unclear. In particular, data on the impact of cryopreservation on the flow cytometric detection of immune escape-associated markers are still scarce [12].

In this study, we compared marker expression across four paired conditions (PB fresh vs. cryopreserved, BM fresh vs. cryopreserved, fresh PB vs. BM, and cryopreserved PB vs. BM) using both MFI and RFI. We chose to primarily interpret results based on RFI, as it represents a background-normalized measure of fluorescence intensity [28].

A key observation is that fresh PB and fresh BM samples did not differ in RFI for any analyzed marker. At the cohort level, this suggests limited systematic compartment-related differences under freshly processed conditions for the markers examined here. However, it should not be interpreted as evidence that PB and BM measurements are interchangeable in individual patients. Paired-line plots showed substantial interindividual variability, and CCC estimates ranged from −0.397 to 0.816, with wide confidence intervals for several marker-condition comparisons (Supplementary Table S2).

After correction for multiple comparisons, no fresh-versus-cryopreserved RFI differences remained statistically significant within PB or BM. By contrast, cryopreserved PB versus BM retained significant RFI differences for CD70 and ULBP1-6. Taken together, the adjusted results indicate limited marker-specific compartment differences after cryopreservation rather than a uniform effect across markers.

The MFI-based analysis showed a clearer cryopreservation-related pattern. In PB, MFI remained significantly higher after cryopreservation for CD184, MICA/B, and ULBP1-6. In BM, adjusted significance remained for CD184 and ULBP1-6, whereas MICA/B did not retain significance. No MFI differences between PB and BM remained after adjustment. These findings are consistent with previous studies demonstrating that cryopreservation and especially the freeze–thaw process can alter the detectability of certain surface markers [5,6,8,12].

The present study was not designed to determine the mechanisms underlying the observed marker-specific differences in antigen detectability. Potential explanations include freeze–thaw-related changes in membrane integrity, receptor internalization or shedding, conformational epitope changes, altered antibody accessibility, and selective loss of blast subpopulations. Differences in cellular composition or compartment-specific microenvironmental regulation may also have contributed. However, these explanations remain hypothetical because no mechanistic or functional validation was performed.

Importantly, the comparison between MFI and RFI still supports the practical value of RFI as a background-normalized parameter. While RFI did not eliminate all differences, it relates marker-associated fluorescence to the corresponding reference signal and thereby accounts for background fluorescence and sample-dependent autofluorescence [29,30]. These findings suggest that RFI provides a complementary, background-normalized measure of marker expression, even though it does not fully compensate for pre-analytical effects related to compartment and cryopreservation.

Notably, pronounced interindividual heterogeneity was observed across all graphs. Paired-line plots frequently displayed non-parallel connecting lines, indicating that patients differed considerably in both the magnitude and direction of condition-related changes in antigen detectability. Although the adjusted analysis identified two statistically significant RFI comparisons, these effects were not uniform across patients. Given the limited cohort size, a small number of pronounced paired differences may therefore have contributed substantially to some group-level findings, which should be interpreted cautiously. To further characterize the potential consequences of this within-patient variability, we applied the predefined conventional 20% reference threshold. This exploratory analysis identified discordant threshold-based classification in 5.3–38.9% of paired measurements, depending on the marker, sample compartment, and processing condition. Although this reference threshold has not been clinically validated for the investigated markers and the analysis does not establish analytical agreement, it illustrates that pre-analytical variation may alter dichotomized marker classification in individual patients. Sensitivity analyses at 10% and 30% supported the overall observation that paired classification can differ between conditions, while the frequency and marker-specific pattern of discordance varied with the selected reference threshold. This threshold dependence further supports interpreting the 20% analysis as illustrative rather than as evidence for a fixed clinical cutoff.

Conversely, mixing different sample types or processing conditions within one patient’s serial measurements (e.g., initial BM fresh, later PB cryopreserved) may introduce condition-related variation that is difficult to distinguish from true biological change. Although the downstream consequences were not directly assessed in the present study, this issue may be particularly relevant in translational and multicenter AML studies using samples stored in a biobank or collected longitudinally, where condition-related shifts in antigen detectability could complicate patient stratification or therapeutic target classification [6,15]. The immediate relevance therefore pertains to translational research rather than routine decision-making. Multicenter biomarker and immunotherapy studies, including assessments of immune checkpoints, NK-cell and CAR-T-cell targets, longitudinal immune monitoring, and measurable residual disease, should use harmonized workflows and meticulously document specimen source and processing.

Several limitations should be considered. This exploratory single-center study included 19 patients. The cohort size may have limited the precision of the estimates and the ability to detect small effects; it did not permit robust subgroup analyses. However, the matched within-patient design reduced interindividual confounding. In addition, procedural variability was minimized by performing cryopreservation and thawing according to the same standardized internal SOP of our institutional biobank and by using the same reagents and handling steps for all cryopreserved aliquots. The study focused on analytical comparability; functional assays and clinical outcomes were outside its scope and should be addressed in future studies. Storage was limited to 24–48 h at −80 °C, and transferability to long-term storage below −150 °C or other cryopreservation protocols remains to be established. Finally, the findings require confirmation in larger, independent AML cohorts and evaluation in other hematologic malignancies.

## 5. Conclusions

Following correction for multiple comparisons, significant RFI differences were restricted to CD70 and ULBP1-6 between cryopreserved PB and BM; no RFI differences remained for comparisons between fresh and cryopreserved samples within either compartment or between fresh PB and BM. Notable MFI changes after cryopreservation persisted for CD184 and ULBP1-6 in both PB and BM and for MICA/B in PB, whereas MFI differences between PB and BM did not remain significant. The exploratory reference-threshold analysis indicated that paired measurements could vary across the predefined 20% threshold. Consequently, sample compartment and processing conditions should remain consistent whenever possible, especially when applying predefined thresholds or assessing serial measurements.

## Figures and Tables

**Table 1:** Proportion of pairs with discordant classification relative to the 20% reference threshold across conditions. Fraction of paired measurements falling on opposite sides of the predefined conventional 20% reference threshold for CD34, CD44, CD70, CD80, CD184, MICA/B, and ULBP1-6 across the four comparisons. Values are reported as discordant/evaluable pairs (percentage).

Marker	PB vs. BM (Fresh)	PB vs. BM (Cryopreserved)	Fresh vs. Cryopreserved (PB)	Fresh vs. Cryopreserved (BM)
CD34	4/19 (21.1%)	2/19 (10.5%)	4/19 (21.1%)	4/19 (21.1%)
CD44	1/19 (5.3%)	1/19 (5.3%)	0/19 (0.0%)	0/19 (0.0%)
CD70	0/18 (0.0%)	0/18 (0.0%)	0/18 (0.0%)	0/18 (0.0%)
CD80	1/18 (5.6%)	0/18 (0.0%)	1/18 (5.6%)	0/18 (0.0%)
CD184	4/19 (21.1%)	4/19 (21.1%)	3/19 (15.8%)	3/19 (15.8%)
MICA/B	3/18 (16.7%)	2/18 (11.1%)	7/18 (38.9%)	6/18 (33.3%)
ULBP1-6	4/18 (22.2%)	5/18 (27.8%)	4/18 (22.2%)	5/18 (27.8%)

## Data Availability

Study data are available to reviewers in Cytobank under the project title “Sample Source and Cryopreservation Affect Flow-Cytometric Profiling of Immune-Escape-Related Markers on AML Blasts Access for Review”.

## Supplementary Materials

The following supporting information can be downloaded at: https://doi.org/10.5281/zenodo.21710841.

## Abbreviations

The following abbreviations are used in this manuscript:

AML	Acute myeloid leukemia
BM	Bone marrow
FMO	Fluorescence minus one
MFI	Median fluorescence intensity
PB	Peripheral blood
PBMC	Peripheral blood mononuclear cells
RFI	Relative fluorescence intensity
CCC	Concordance correlation coefficient
